# Trabectedin may be a valuable treatment option for elderly patients with metastatic soft tissue sarcomas

**DOI:** 10.3389/fonc.2024.1437732

**Published:** 2024-07-25

**Authors:** Gianmaria Miolo, Angela Buonadonna, Davide Lombardi, Simona Scalone, Andrea Lauretta, Lara Della Puppa, Giuseppe Corona

**Affiliations:** ^1^ Medical Oncology and Cancer Prevention Unit, Centro di Riferimento Oncologico di Aviano (CRO), IRCCS, Aviano, Italy; ^2^ Unit of General Oncologic Surgery, Centro di Riferimento Oncologico di Aviano (CRO), IRCCS, Aviano, Italy; ^3^ Oncogenetics and Functional Oncogenomics Unit, Centro di Riferimento Oncologico di Aviano (CRO), IRCCS, Aviano, Italy; ^4^ Immunopathology and Cancer Biomarkers Unit, Centro di Riferimento Oncologico di Aviano (CRO), IRCCS, Aviano, Italy

**Keywords:** cancer, trabectedin, elderly, sarcoma, first-line treatment, pharmacokinetics

## Abstract

**Background:**

In the landscape of metastatic soft tissue sarcoma (mSTS) treatment, anthracyclines have shown efficacy; however, their associated toxicity imposes significant limitations, especially in frail elderly patients with mSTS who are highly susceptible to severe adverse effects. In this context, trabectedin, due to its distinct pharmacological profile and safety profile, may represent an interesting alternative being demonstrated to be active in treating mSTS. These features hold particular significance for elderly and unfit patients with mSTS, where balancing treatment benefits with potential adverse effects represents the pivotal objective.

**Methods:**

The investigation was focused on a specific group of 11 elderly patients with mSTS aged ≥70, all undergoing first-line treatment with trabectedin, and it was supported by comprehensive pharmacokinetic and pharmacodynamic studies. Among these patients, 9 out of 11 started the treatment at a dose of 1.5 mg/m^2^.

**Results:**

The primary objective of this investigation is to highlight trabectedin as a valuable first-line treatment option for elderly and unfit patients with mSTS. Additionally, this investigation seeks to explore whether higher administered doses of trabectedin can enhance clinical outcomes while maintaining the same toxicity profiles. The median progression-free survival (PFS) was 77 days (95% CI, 53–89), the median overall survival (OS) was 397 days (95% CI, 66–2,102), while the overall toxicity of grade 3–4 severity amounted to 43%.

**Conclusion:**

These findings provide new insights into the clinical outcomes and toxicity associated with trabectedin in an elderly patient population, enhancing our understanding of better treatment approaches for a specific population of patients with mSTS.

## 1 Introduction

The choice of the most suitable treatment options for patients with locally advanced or metastatic soft tissue sarcoma (mSTS) is a complex task that requires a comprehensive understanding of both the tumor histological–molecular characteristics and the clinical conditions of the patient. This challenge is significantly heightened for the elderly mSTS population where common age-related dysfunctions as well as the frequent presence of comorbidities, which are important signals of a fragile health status, pose a significant obstacle in tailoring an effective healthcare approach (1–4). The natural aging process contributes to a decline in essential organ functions, particularly the liver and kidneys wherein altering the clearance drugs can have a detrimental effect on their safety profile (5, 6). In this context, considering the elevated risk of toxicity linked to traditional cytotoxic agents (7–9), there is a pressing need to explore innovative treatments capable of ensuring in elderly population active treatments with favorable safety profiles.

A subgroup analysis of patients over 65 years revealed that single-agent doxorubicin yielded an overall survival (OS) of 9.8 months (95% CI, 7.4–11.5), which was comparable to the OS of 9.9 months (95% CI, 5.9–11.8) for those treated with epirubicin over a 24-month follow-up while trabectedin demonstrated an OS of 17.3 months (95% CI, 9.4–17.3) despite a shorter 6.7-month follow-up (10). In a different study involving a large cohort of 361 elderly patients with mSTS, anthracycline-based regimens achieved a median OS of 10.9 months, but 32% of patients experienced severe hematological toxicities that required treatment discontinuation in 16% of them (7). Analogously, in a phase II study involving 40 elderly patients with mSTS aged 60 to 84 years (median age, 70.5 years) treated with doxorubicin, the OS was 9.8 months (95% CI, 6.7–11.6), alongside a notable severe side effect rate of 59% (11).

When extending the age threshold to ≥70 years, the anthracycline-based chemotherapy conferred survival advantage over best supportive care but did not demonstrate a survival advantage compared to other treatments. Moreover, it led to a significant grade 3–4 toxicity rate (ranging from 33% to 58%), further underscoring the challenges in managing these patients (9).

Trabectedin is an antineoplastic agent primarily indicated for the treatment of patients with mSTS following the failure of anthracycline-based regimens that presents a multifaceted mechanism of action such as targeting DNA interactions, transcriptional processes, and DNA repair mechanisms (12–16). One of the distinct advantages of trabectedin is its well-tolerated safety profile characterized by adverse events that are generally reversible and noncumulative (17–20). Thus, these pharmacological features may make it a valuable choice in the population of elderly and unfit patients with mSTS, especially where the primary objective is a viable treatment option preserving an acceptable quality of life (21). A previous clinical exploration performed on elderly patients with mSTS seems to indicate promising outcomes for this drug when utilized as first-line treatment, showing a median progression-free survival (PFS) of 4 months and an OS of 12 months, respectively (22). Recently, a multicentric study aimed at evaluating the feasibility and prognostic value of comprehensive geriatric assessment investigated 69 patients with STS, 56 of whom were aged ≥70 years, obtaining a PFS of 2.5 months and an OS of 11.2 months (23). Especially for the leiomyosarcoma histotype, it was observed that frontline treatment based on the combination of doxorubicin and trabectedin could lead to a doubling of PFS compared to doxorubicin alone (12.2 months vs. 6.2 months) (24).

While these results are promising, they require further confirmation to ascertain the possible alternative option of trabectedin as a first-line treatment in an elderly population of patients with mSTS.

The objective of the current study is to advance existing research by investigating the use of trabectedin as a first-line treatment for elderly patients with mSTS, aiming to determine whether higher administered doses of trabectedin can enhance clinical outcomes while maintaining the same toxicity profiles.

This is achieved by presenting a monocentric experience, detailing a comprehensive pharmacokinetic and pharmacodynamic investigation conducted on a specific group of 11 elderly patients with mSTS aged ≥70, all undergoing first-line treatment with trabectedin. This clinical pharmacology exploration provides further valuable insights into the clinical outcomes and toxicity associated to trabectedin in this specific demographic, thereby enhancing our understanding of treatment strategies for elderly patients with mSTS.

## 2 Materials and methods

### 2.1 Clinical population

All patients with a histological diagnosis of locally advanced or mSTS (25) who met the following criteria were consecutively enrolled in a clinical trial aimed at identifying pre-dose plasma metabolomics signatures potentially associated with individual variations in trabectedin pharmacokinetics: normal hematological, renal (≤1.6 mg/dL), liver, and cardiac functions; a performance status (PS) ≤ 2; no CNS metastases; or no history of previous cancer. All patients aged 70 or older who were considered unsuitable for standard first-line treatment with anthracycline-based regimens and underwent first-line treatment with trabectedin were evaluated. This prospective, monocentric clinical investigation focused on 11 consecutive elderly patients. Among these, 4 had received neoadjuvant radiotherapy and 7 had undergone surgical treatment. Only two patients received adjuvant treatments consisting of radiotherapy or chemotherapy, respectively. The surgical interventions resulted in no residual disease (R0) in three cases, microscopic residual disease (R1) in three cases, and macroscopic residual disease (R2) in the remaining case.

Trabectedin was administered to the patients at a dosage ranging from 1.1 to 1.5 mg/m^2^ with a maximum of dose of 2.6 mg per cycle, infused over 24 h every 3 weeks via a central venous catheter.

A baseline CT scan was conducted at the onset of treatment, followed by another scan after 12 weeks. In patients showing no progression, tumor imaging assessments were continued every 3 months for an additional year, and then reduced to once every 6 months.

Treatment continued until disease progression or development of intolerable adverse events that required treatment discontinuation. Dexamethasone premedication at 4 mg twice a day, beginning the day before trabectedin administration and continuing for two consecutive days after with 4 mg a day, was administered to all patients. Response assessment was conducted in accordance with the Response Evaluation Criteria in Solid Tumors (RECIST) version 1.1 while the toxicity was reported according to CTCAE version 3. Each patient provided informed consent for participation in the investigation. This clinical investigation adhered to the principles outlined in the Declaration of Helsinki and followed the Good Clinical Practice Guidelines of the International Conference on Harmonization. The study protocol was subjected to review and approval by the institutional review board.

### 2.2 Pharmacokinetics study

For the pharmacokinetics analysis, blood samples were systematically collected at various time points during the trabectedin infusion and its elimination phases over 48 h. The sampling schedule included pre-dose (before administration) and during infusion at 2, 8, and 24 h (end of infusion), whereas post-infusion samples were obtained at 0.5, 1, 4, 8, and 24 h. Plasma concentration of trabectedin at these time points was measured by the high-performance liquid chromatography with tandem mass spectrometry (LC/MS/MS) method (26). The lower limit of quantification for this method was 0.01 ng/mL, providing a reliable range for detecting and measuring trabectedin concentrations in the collected samples. Pharmacokinetic parameters were calculated from the drug plasma concentration vs. time profiles using a non-compartmental model (27). The area under the curve up to 48 h (AUC0–48) was calculated using the trapezoidal method, the area under the moment curve (AUMC0–48) was calculated as the (concentration • time) time data plot, the mean resident time (MRT) was calculated as the AUC0–48/AUMC0–48 ratio, ***t*_1/2_** was calculated from 0.693/***k*** where ***k*** is the slope of the late phase of the logC vs. time curve, while the drug clearance was estimated by dose/AUC0–48. The C_max_ and C_last_ were derived from the pharmacokinetics profile and corresponded to the concentration of trabectedin at the end of the 24-h infusion and at 48 h from the start of infusion.

### 2.3 Statistical methods

Survival curves were generated using the Kaplan–Meier method, and data analyses were carried out using MedCalc Statistical Software for Windows, version 19.4 (MedCalc Software bv, Ostend, Belgium; https://www.medcalc.org; 2020).

## 3 Results

The median age of the patients at study entry was 76 years [interquartile range (IQR), 75–79 years; minimum–maximum range of 70–90 years]. The Eastern Cooperative Oncology Group (ECOG) performance status reflected as 0 in five patients, 1 in five patients, and 2 in the remaining patient. Predominantly, leiomyosarcoma and liposarcoma constituted the most frequent histological subtypes (55%) followed by pleomorphic sarcoma (18%). High-grade tumors were prevailing, representing 82% (9 out of 11) of the cases (**Table 1**).

**Table 1:** Characteristics of the enrolled patients and their tumors.

Age	Mean (SD)	78	5.5
	Median (IQR)	76	75–79
Sex	Female	3	73%
	Male	8	27%
ECOG	0	5	45%
	1	5	45%
	2	1	10%
BMI	Mean (SD)	27	4.0
	Median (IQR)	27	23.1–31.4
Grade	2	2	18%
	≥3	9	82%
Histotype	Leiomyosarcoma^	3	27%
	Liposarcoma*	3	27%
	Others°	5	46%
Primary site of disease	Retroperitoneum	4	36%
	Gluteus	3	27%
	Trunk	3	27%
	Forearm	1	10%
Metastases			
	Lung	6	55%
	Extra-lung	5	45%
Median trabectedin courses (IQR)		3	3–6

^ One pleomorphic leiomyosarcoma (PLMS) and two leiomyosarcoma NAS.

* One well-differentiated liposarcoma (WDLS), one dedifferentiated liposarcoma (DDLS), and one myxoid liposarcoma (MLS).

° Three undifferentiated pleomorphic sarcoma (UPS), one myxofibrosarcoma, and one sarcoma NAS.

In the overall population, the median number of trabectedin courses administered was 3 (IQR, 3–6), with four patients (29%) receiving six or more cycles. Specifically, patients with L-sarcoma received a median of 4.5 courses (range, 1–21), while patients with Other-sarcoma received a median of 3 courses (range, 3–6). The starting dose of trabectedin was set at 1.5 mg/m² for nine patients, while in two cases, the dose administered was 1.1 mg/m², chosen by a physician based on frailty characteristics, including performance status and multiple comorbidities. Treatment discontinuation primarily occurred due to disease progression (PD), followed by medical decision. One patient died 33 days after treatment initiation and was subsequently excluded from the response assessment. After the third cycle, PD was observed in six patients (60%), while four patients (40%) demonstrated disease stability (SD). Among the histological subtypes responsive to treatment, four were from the L-sarcoma group, while the remaining responsive case belonged to the Other-sarcoma group, specifically a pleomorphic sarcoma.

**Figure 1:**
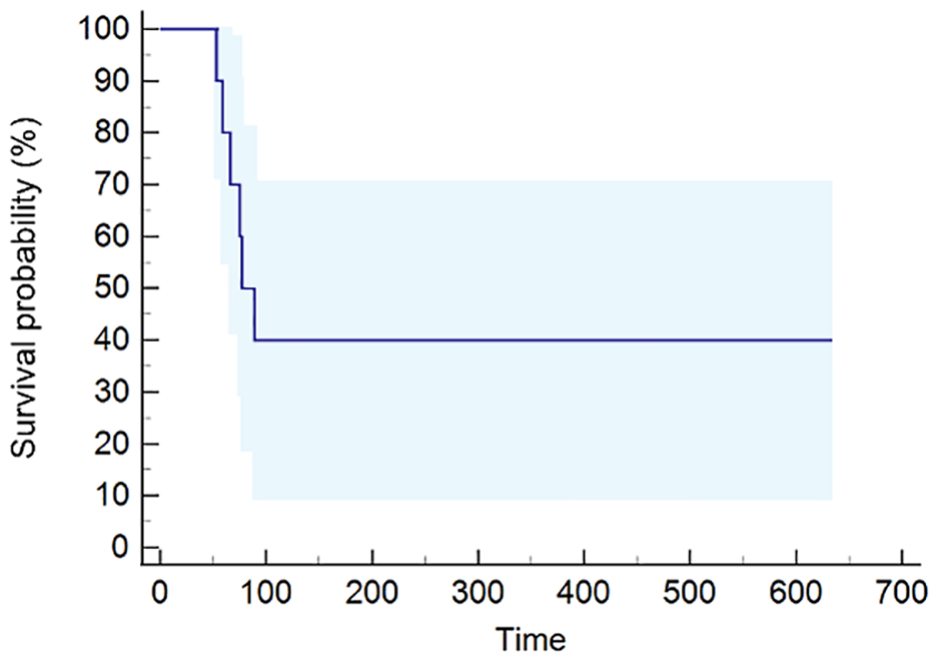
Progression-free survival (PFS) curve calculated using the Kaplan–Meier method.

**Figure 2:**
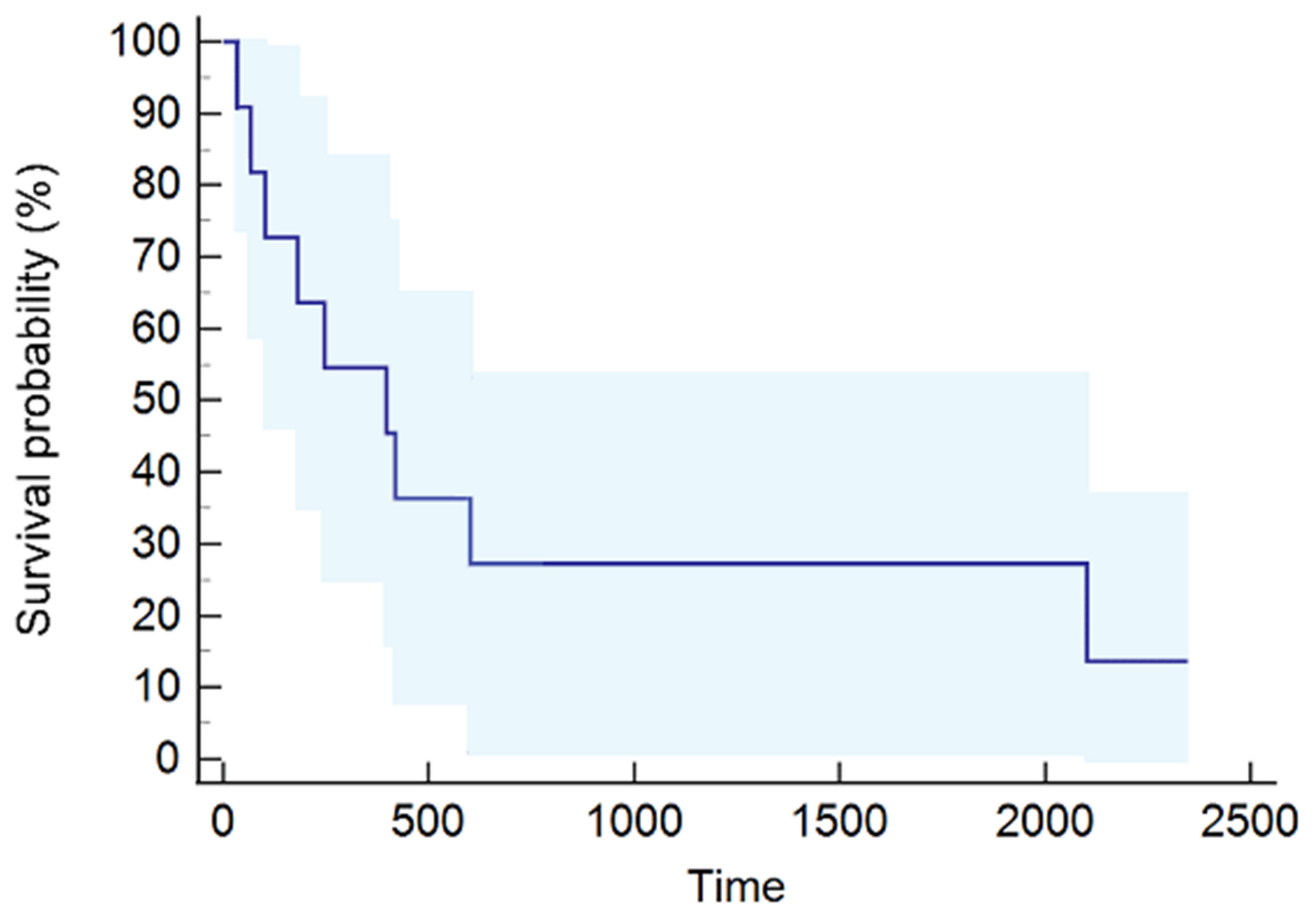
Overall survival (OS) curve calculated using the Kaplan–Meier method.

The median PFS was 77 days (95% CI, 53–89) (**Figure 1**), while the median OS was 397 days (95% CI, 66–2,102) (**Figure 2**). The median follow-up was 2.11 years, ranging from 0.1 to 7.66.

A total of 14 adverse events were documented, with six of them (43%) classified as grade 3–4. Specifically, hematological toxicity of grade 3–4 was observed in 29%, while non-hematological grade 3 toxicity accounted for 14%. Among hematological toxicities, neutropenia was the most prevalent, followed by leukopenia, while among non-hematological toxicities, emesis emerged as the most frequent (**Table 2**). Overall, five patients (55%) required a reduction in their initial treatment dose. Among them, two patients underwent dose reduction starting from the second cycle, while the remaining three, including one who initially started with a reduced dose, began the reduction from the fourth cycle onwards (**Table 2**).

**Table 2:** Hematological and non-hematological toxicities.

n	Dose*	Toxicity NE		Toxicity E		First reduction	Second reduction
		G1–G2	G3–G4	G1–G2	G3–G4		
1	2.6	–	G3	–	G3	From fourth cycle to 1.9 mg	
2	2.6	–	–	–	G4		
3	2.6	–	–	G1	–		
4	2.6	G1	–	–	–	From second cycle to 1.6 mg	
5	2.6	–	–	–	G4		
6	2.4	–	–	G2	–		
7	1.8	G2	–	G2	–		
8	2.6	G2	–	G2	–	From fourth cycle to 2.5 mg	From fifth cycle to 2.3 mg
9	2.6	–	G3	–	G3	From second cycle to 2.2 mg	From third cycle to 1.9 mg
10	2.0	G2	–	–	–	From fourth cycle to 1.9 mg	
11	2.6	–	–	–	–		
		4/11 (36%)	2/11 (18%)	4/11 (36%)	4/11 (36%)		

*Total dose (mg).

Overall, 6 of these 11 patients received a second-line treatment consisting of gemcitabine plus dacarbazine (4), eribulin (1), and doxorubicin single-agent regimen (1). Only three of these patients, previously treated with the combination regimen, received a third-line treatment with pazopanib (2) and eribulin (1).

**Figure 3:**
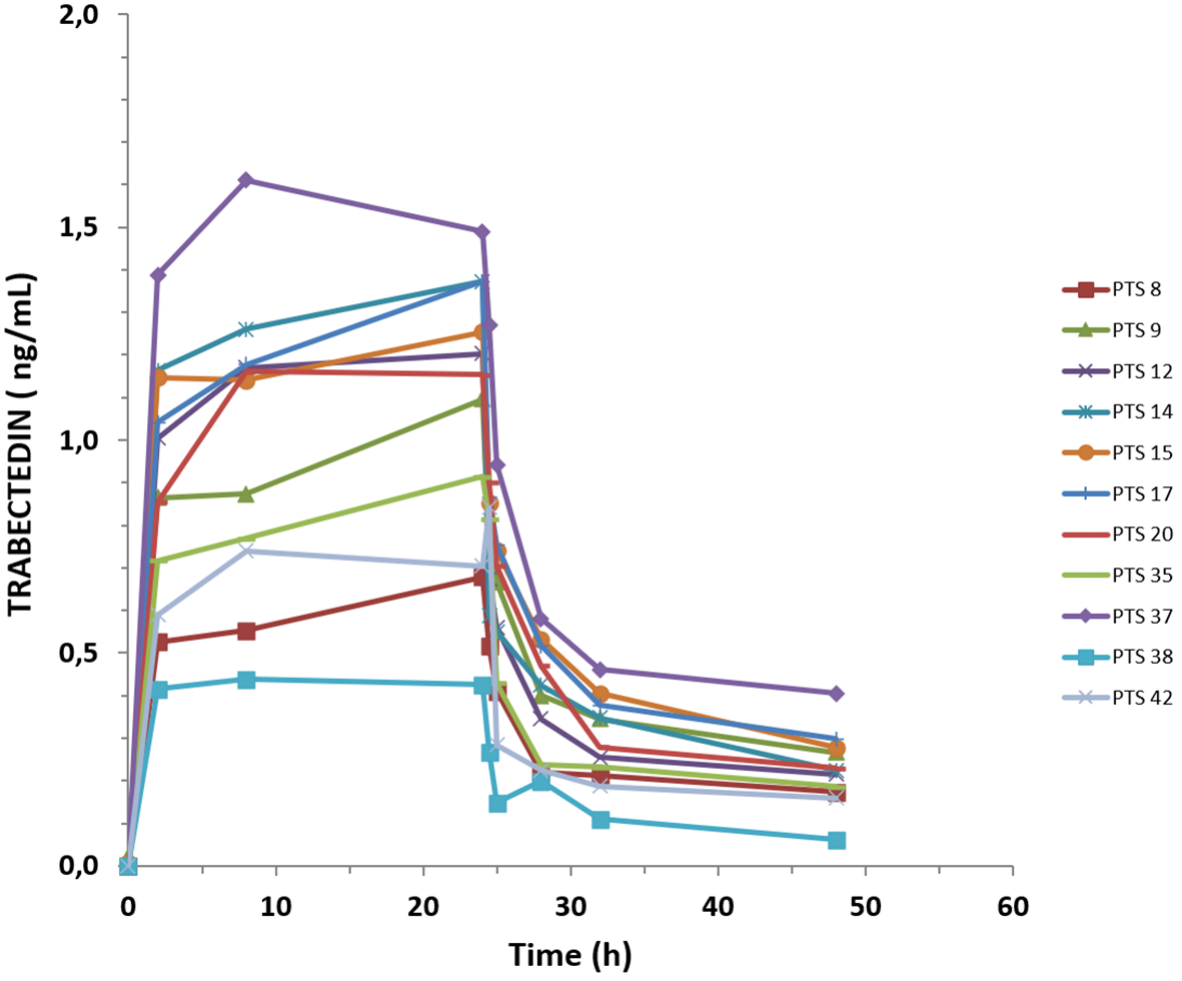
Plasma concentration–time profile of trabectedin detected in the 11 analyzed patients.

Pharmacokinetic assessments were conducted during the first cycle of treatment for all patients. The mean dose of trabectedin administered was 1.32 mg/m^2^ (SD ± 0.15). **Table 3** shows the characteristics of each enrolled patient, and **Figure 3** summarizes the plasma pharmacokinetics profile of trabectedin in all patients investigated. At approximately 8 h of infusion and up to the end of the 24-h infusion, trabectedin reached and maintained steady-state concentration, followed by a rapid decline within 6 h from the end of infusion according to multiphase elimination steps. At 24 h from the end of the infusion, trabectedin was still measurable in all patients, and the mean concentration at 48 h (C_last_) was 0.2 ng/mL (IQR, 0.2–0.3; minimum–maximum range, 0.1–0.4). The other mean pharmacokinetics parameters of trabectedin were as follows: the drug concentration evaluated at the end of 24 h of infusion, expressed as C_max_, is 1.1 ng/mL (IQR, 0.8–1.4; minimum–maximum range, 0.4–1.6); the trabectedin exposure expressed as AUC0–48h is 30.6 ng/mL*h (IQR, 21.2–37.9; minimum–maximum range 12.7–47.7); estimated clearance = 48.6 L/h/m^2^ (IQR, 36.6–60.2; minimum–maximum range, 27.2–87.7), and the mean residence time (MRT) = 18.1 h (IQR, 17.5–18.7; minimum–maximum range 16.9–19.3) (**Table 4**). These parameters did not differ significantly from those observed in a group of 31 patients with age ≤65 years undergoing second-line treatment and are superimposable with those observed in previously pharmacokinetic investigations (**Supplementary Table 1**).

**Table 3:** Physical and biochemical characteristics of each enrolled patient.

PTS (*n*)	Sex	Age (years)	Weight (k)	Height (cm)	BSA (m^2^)	Serum creatinine (mg/dL)	Creatinine clearance (mL/min)*	BT (mg/dL)	BD (mg/dL)	AST (U/L)	ALT (U/L)	Hb (g/dL)
1	M	75	74	165	1.8	0.82	81.5	1.54	0.78	16	11	13.7
2	M	71	96	173	2.1	1.59	57.9	0.63	0.27	25	20	13.8
3	M	90	83	173	2.0	1.00	57.6	0.34	0.14	11	9	11.9
4	M	77	59	167	1.7	1.32	39.1	1.47	0.15	19	17	14.1
5	F	79	75	168	1.9	0.73	74.0	0.69	0.52	33	26	13.2
6	F	75	73	146	1.6	1.00	56.0	0.28	0.30	17	11	10.5
7	F	88	60	165	1.7	0.79	46.6	0.30	0.14	11	8	11.1
8	M	76	79	170	1.9	0.80	87.8	0.59	0.11	20	12	11.2
9	M	75	83	163	1.9	0.96	78.1	0.40	0.20	17	22	13.,1
10	M	79	75	180	1.9	1.04	61.1	0.50	0.21	9	6	12.2
11	M	75	82	183	2.0	1.07	69.2	0.50	0.20	24	24	14.8

* The creatinine clearance was calculated using the Cockroft–Gauilt equation.

**Table 4:** Pharmacokinetic parameters of trabectedin.

	C_max_ (ng/mL)	AUC_0–48_ (ng/mL*h)	MRT (h)	CL (L/h/m^2^)	C_last(48h)_ (ng/mL)
Mean	1.1	30.6	18.1	48.6	0.2
SD	0.3	10.2	0.8	18.7	0.1
Median	1.2	33.6	18.1	44.0	0.2
Min	0.4	12.7	16.9	27.2	0.1
Max	1.6	47.7	19.3	87.7	0.4
*n*	11	11	11	11	11

## 4 Discussion

While anthracyclines have proven to be effective in mSTS treatment, their notable toxicity poses challenges in administering this class of drugs to frail elderly patients with cancer who are more susceptible to adverse events (7, 9–11). Trabectedin emerged as a compelling therapeutic alternative for this specific patient population due to its favorable safety profile, characterized by a reduced incidence of both non-hematological and hematological toxicity (28, 29).

Recent studies have emphasized the potential role of systemic inflammatory indices, such as the lower lymphocyte/monocyte ratio, in predicting trabectedin efficacy in frail elderly patients with STS, further highlighting the importance of these biomarkers in treatment planning (30–33) of elderly patients. Thus, the tumor-related systemic inflammation, coupled with its favorable toxicity, make trabectedin a possible alternative to anthracyclines in elderly patients with mSTS. In this context, our monocentric study aims to assess the effectiveness of trabectedin as tailored frontline treatment in a cohort of elderly patients with mSTS aged ≥70 years.

This age threshold exceeds the conventional definition of elderly patients, typically set at 65 years, but the extension of this age threshold acknowledges the evolving landscape of cancer patient demographics, thus providing a more accurate representation of the elderly population observed in clinical practice.

In order to provide key information on the optimal drug dosage, administration frequency, and potential dose adjustments, pharmacokinetic investigations have been performed in all patients enrolled in the study. The mean clearance of trabectedin was 48.6 L/h/m^2^ (SD ±18.78), slightly higher than values reported in other studies (34–38), although not significantly different from the value of 39.98 L/h/m^2^ (SD ±14.08) reported in a study conducted in a highly selected population of patients with mSTS aged ≥65 years (22). These results appear in contrast with the diminished organ functionality, gradual decline in liver volume, and altered expression profile of CYP3A4, typically observed in older patients and potentially leading to a reduction in the metabolic clearance of trabectedin (6, 39–41). However, it is worth noting that in managing elderly patients with mSTS, a concurrent administration of dexamethasone is employed to achieve an optimal balance between drug metabolism, efficacy, and adverse events (42). Dexamethasone is known to act as a CYP3A4 concentration-dependent inducer playing a pivotal role in increasing the metabolic clearance of trabectedin and reducing drug-induced hepatotoxicity and myelosuppression (43). Interestingly, no significant alteration in the main drug metabolism emerged in our selected patients, despite the fact that reduced CYP3A4 expression has been observed and dexamethasone has a decreased capacity to induce CYP3A4 in the elderly population (6, 44, 45). The pharmacokinetic findings emphasize that elderly patients receiving 1.5 mg/m^2^ of trabectedin, along with dexamethasone premedication, maintain an effective trabectedin clearance comparable to younger patients who received trabectedin as a second-line treatment. Indeed, the inter-patient variability of AUC0–48h, which is a surrogate of total drug exposure for elderly patients, was similar to that observed in a group of 31 patients with mSTS aged ≤70 years undergoing second-line treatment (**Supplementary Table 1**). AUC0–48h was found to be not correlated with toxicity, indicating that pharmacokinetic profiles are more comprehensively influenced by a combination of multiple factors including individual genetics, specific physio-pathological conditions, and environmental variables rather than being solely determined by age (46).

Beyond a favorable toxicity safety profile, this exploratory assessment revealed a notable clinical benefit of 40%, indicating trabectedin as an interesting treatment choice for this group of patients who often has limited therapeutic alternatives. Although the overall clinical benefit was 50% lower than that reported by Grosso et al. (22), it is worth noting that the latter included fewer cases with unfavorable prognosis. Indeed, our elderly population was characterized by 45% of cases with Other-sarcoma, while in the previous study, where a clinical benefit that is twofold higher was achieved, only 20% of the patients were classified as Other-sarcoma, with a net predominance of L-sarcoma. Thus, the different percentages of L-sarcoma that, according to the literature, represent the histological subtypes most responsive to trabectedin treatment can be partially responsible for this apparent incongruity observed between the two studies. Despite this heterogeneity, median PFS and OS were found to be approximately 3 and 12 months, respectively, suggesting that trabectedin, when used as a first-line treatment in a population of elderly patients with mSTS, has no negligible clinical impact in view of the fact that such clinical outcomes were reached using a starting dose of 1.3 mg/m^2^ of trabectedin. Overall, the clinical outcomes observed in this investigation, as well as in the previous study of Grosso et al. (22), did not significantly differ from those previously reported in patients with mSTS aged 60 years or older who were randomized to receive pazopanib or doxorubicin as first-line treatment (47), with the PFS ranging between 4.4 and 5.3 months across treatment arms and the OS ranging between 12.3 and 14.3 months, while the adverse events of grade 3–4 severity amounted to 85.6%.

Although the small number of patients limits the ability to draw definitive conclusions, the results of this investigation provide valuable confirmation of previous findings (22, 23) contributing to establishing a more solid foundation for the use of trabectedin treatment in elderly patients with mSTS.

## 5 Conclusion

Optimizing the management of STS in elderly patients is a significant clinical challenge, particularly because this demographic represents a substantial and growing proportion of the cancer population due to increasing life expectancy. The findings of this study seem to confirm that 1.3 mg/m^2^ dose of trabectedin represents a valuable first-line pharmacological option for treating elderly patients with mSTS, given its favorable balance between clinical efficacy and lower toxicity profile that directly affects quality of life.

## Data availability statement

The original contributions presented in the study are included in the article/**Supplementary material**. Further inquiries can be directed to the corresponding author.

## Ethics statement

The studies involving humans were approved by Institutional Review Board of CRO Aviano. The studies were conducted in accordance with the local legislation and institutional requirements. Written informed consent for participation in this study was provided by the participants’ legal guardians/next of kin.

## Author contributions

GM: Conceptualization, Writing – original draft, Writing – review & editing. AB: Writing – review & editing. DL: Writing – review & editing. SS: Writing – review & editing. AL: Writing – review & editing. LDP: Writing – review & editing. GC: Conceptualization, Writing – original draft, Writing – review & editing.
