# Supplementary material for: Trabectedin may be a valuable treatment option for elderly patients with metastatic soft tissue sarcomas
Source: Front Oncol. 2024 Jul 25;14:1437732. doi: 10.3389/fonc.2024.1437732 (PMC11306058; doi:10.3389/fonc.2024.1437732)
Supplement: Supplementary file 1 [file Table_1.docx]

TABLE 1S Pharmacokinetic parameters of the 31 enrolled patients undergoing first-line treatment

|  | C_max_ ng/mL | AUC 0-48 ng/mL*h | MRT(h) | *CL (L/h/m^2^)* | *C_last(48h) ng/mL_* |
| --- | --- | --- | --- | --- | --- |
| Mean | 1.3 | 34.5 | 17.7 | 43.8 | 0.2 |
| SD | 0.7 | 11.7 | 3.1 | 17.1 | 0.1 |
| Median | 1.2 | 34.8 | 18.1 | 40.7 | 0.2 |
| Min | 0.3 | 19.3 | 0.8 | 18.7 | 0.1 |
| Max | 4.6 | 63.4 | 23.1 | 87.7 | 0.5 |
| N | 31 | 31 | 31 | 31 | 31 |
